# Vascular endothelial growth factor promotes the expression of cyclooxygenase 2 and matrix metalloproteinases in Lewis lung carcinoma cells

**DOI:** 10.3892/etm.2012.702

**Published:** 2012-09-10

**Authors:** JIANWU HU, CAIYUN CHEN, YUAN SU, JIAO DU, XIN QIAN, YANG JIN

**Affiliations:** Department of Pulmonary Medicine, Union Hospital, Tongji Medical College, Huazhong University of Science and Technology, Wuhan 430022, P.R. China

**Keywords:** lung neoplasm, vascular endothelial growth factor, cyclo oxygenase-2, metalloproteinases

## Abstract

Vascular endothelial growth factor (VEGF) plays a critical role in tumor progression, angiogenesis and metastasis. Cyclooxygenase (COX)-2, matrix metalloproteinase (MMP)2, MMP9 and wild-type (WT) p53 has been found to regulate the production of VEGF. Whether VEGF regulates the production of COX-2, MMP2, MMP9 and WTp53, however, has yet to be determined. This study examined the influence of the overexpression or knockdown of VEGF on the protein levels of COX-2, MMP2, MMP9 and WTp53 as well as cell growth and cell cycle progression in Lewis lung carcinoma (LLC) cells. LLC cells were transfected with pIRES2-VEGF-GFP in the VEGF-overexpressing group (LLC-VEGF), pIRES2-GFP in the mock group (LLC-GFP) or pSUPER-VEGF-GFP in the VEGF knockdown group (LLC-RNAi). Protein levels were detected by western blot analysis. LLC cell growth exhibited no marked change in the LLC-VEGF group, but was significantly retarded in the LLC-RNAi group. Further examination revealed that more cells entered the S stage in the LLC-VEGF group than in the control (or mock) group (45.3 vs. 29.1%, P<0.05), and that cell growth was retarded in the LLC-RNAi group. Moreover, COX-2 and MMP2 and MMP9 proteins were significantly increased in the LLC-VEGF group (approximately 1.84-, 1.89- and 1.83-fold, respectively, vs. control, P<0.05), but significantly decreased in the LLC-RNAi group, whereas the expression of WTp53 exhibited the opposite pattern of change. VEGF expression was positively correlated with COX-2, MMP2 and MMP9 expression (r=0.984, r=0.978, r=0.969, respectively, P<0.01) and negatively correlated with WTp53 (r=−0.833, p<0.01). The activities of MMP2 and MMP9 were increased in the LLC-VEGF group. In conclusion, VEGF overexpression may promote the expression of COX-2 and MMPs, but inhibits WTp53 production in LLC cells; VEGF underexpression may have an inverse effect. These changes are closely correlated with the infiltration and metastasis of lung cancer.

## Introduction

Lung cancer was the most commonly diagnosed cancer as well as the leading cause of cancer-related death in males and the second cause of cancer-related death in females globally in 2008 (1). To date, no effective treatment is available and the five-year survival rate remains less than 15%, despite advances in diagnosis and treatment. Numerous patients suffer from recurrence and metastasis following surgery, chemotherapy and radiotherapy (2).

Angiogenesis is crucial for tumor growth and metastasis, and can be stimulated by several regulators including vascular endothelial growth factor (VEGF), basic fibroblast growth factor (bFGF), transforming growth factor-β (TGF-β), platelet-derived growth factor (PDGF), interleukin-8, and angiogenin. Among these, VEGF has the greatest potential for research (3,4). The level of VEGF is significantly increased in NSCLC and is intimately associated with tumor metastasis as well as poor prognosis (5–10).

Cyclooxygenase (COX) is an enzyme that catalyzes the rate-limiting step in prostaglandin synthesis. To date, two isoforms, COX-1 and COX-2, have been identified (11). COX-1 is constitutively expressed in virtually all cells, whereas COX-2 is only expressed in regions of inflammation and in the cancer microenvironment. COX-2 overexpression has been observed in the majority of human malignancies, including lung cancer, and its expression level correlates with VEGF (12–17). Matrix metalloproteinases (MMPs) are a diverse family of enzymes capable of degrading various components of the ECM. MMP expression has been found to be upregulated in several human tumors and correlates with advanced stage, invasion, angiogenesis, metastatic properties and poor prognosis (18). Among secreted MMPs, MMP2 and MMP9 (gelatinase A and gelatinase B) are known to play a key role in tumor invasion and metastasis development (19). The expression of large amounts of MMP2 and MMP9 has been documented in NSCLC (20). Wild-type (WT)p53 is one of the most intensively studied anti-oncogenes. It acts as a nuclear transcription factor that transactivates genes involved in apoptosis, cell cycle regulation, and other vital processes (21). A previous study revealed that WTp53 is significantly inversely associated with VEGF expression (22). On the basis of these studies, it is conceivable that VEGF expression may correlate with COX-2, MMP and WTp53 expression. However, the relationships between VEGF expression and tumor growth and cell cycle status have not been examined previously.

Therefore, the present study aimed to elucidate the relationship between VEGF expression and COX-2, MMP2, MMP9 and WTp53 expression and cell growth and cell cycle progression in Lewis lung cancer (LLC) cells.

## Materials and methods

### Reagents

Lipofectamine 2000™, G418 and propidium iodide were obtained from Invitrogen (Carlsbad, CA, USA). Plasmid mini kit and DNA Gel extraction kit were purchased from Omega Bio-Tek (Norcross, GA, USA). Rabbit anti-mouse VEGF polyclonal antibodies and mouse anti-human COX-2, MMP2, MMP9, phospho-MMP2 and phospho-MMP9, WTp53 and β-actin monoclonal primary antibodies, sheep anti-mouse and sheep anti-rabbit secondary antibodies labeled horseradish peroxidase were all purchased from Santa Cruz Biotechnology Inc. (Santa Cruz, CA, USA). The recombinant plasmids of pIRES2-VEGF-GFP (VEGF overexpression plasmid), pIRES2-GFP and pSUPER-VEGF-GFP (microRNA expression of VEGF plasmid) were kindly provided by Dr Yuan Su.

### Cell culture

LLC, a mouse lung cancer cell line, was purchased from the cell bank of the Chinese Academy of Science, Shanghai, China. Cells were stored in a humidified 5% CO_2_ atmosphere and cultured in 1640 medium supplemented with 10% fetal calf serum, glutamine (2 mM), penicillin (100 U/ml) and streptomycin (100 U/ml).

### Cell transfection and stable cell line establishment

LLC cells were transfected with pIRES2-VEGF-GFP (LLC-VEGF), pSUPER-VEGF-GFP (LLC-RNAi), pIRES2-GFP (LLCGFP) plasmids using Lipofectamine 2000. The stable cells were selected following 2 months in 1 mg/ml of G418-containing medium. The resistant colonies were removed and VEGF protein expression was examined. The selected colonies were separately maintained in 1640 medium supplemented with 10% fetal calf serum, glutamine (2 mM), penicillin (100 U/ml), streptomycin (100 U/ml) and 100 *μ*g/ml G418. After stable expression was achieved, the cells were observed under fluorescence microscopy. Parent cells (LLC) were used as the control group.

### In vitro cell growth assay

To compare the cell growth *in vitro*, cells were seeded into a 96-well plate at a density of 1×10^4^ cells/ml (200 *μ*l per well), and the number of cells of each cell line was counted directly every day. All experiments were repeated three times.

### Flow cytometric analysis of the cell cycle

Cultured cells were harvested after 48 h and washed with phosphate-buffered saline (PBS). The cell cycle status was then analyzed by flow cytometry as described previously. Briefly, 1×10^6^ cells were washed twice with PBS, re-suspended in a buffer (500 *μ*l; containing 0.5% Triton X-100, PBS, 0.05% RNaseA) and incubated for 30 min. Finally, 400 *μ*l of propidium iodide solution (50 mg/ml) and 400 *μ*l Annexin V-FITC were added. Cells were then left on ice for 30 min. Fluorescence emitted from propidium iodide-DNA complexes was quantified after laser excitation of the fluorescent dye by fluorescence-activated cell sorting flow cytometry (Becton Dickinson, Mountain View, CA, USA). The cell cycle distribution and the percentage of apoptotic cells were determined by measuring the DNA content of the cells.

### Western blotting

To prepare the whole cell extracts for western blotting, cells were harvested 48 hours after the culture and washed three times with PBS, and lysed in radio-immunoprecipitation lysis buffer [50 mM Tris-Cl (pH 7.4), 1% Nonidet P-40, 40 mM NaF, 10 mM NaCl, 10 mM Na_3_VO_4_, 1 mM phenylmethylsulfonyl fluoride, 10 mM dithiothreitol, and 1 *μ*g/ml each of leupeptin and aprotinin]. The cell lysates (50 *μ*g of protein) were separated by SDS-PAGE followed by transfer to polyvinylidene difluoride membranes. Size approximations were made by comparing the stained bands with those of the marker or ladder loaded during electrophoresis. After blocking with 10% non-fat dry milk or bovine serum albumin in Tris-buffered saline containing 0.1% Tween-20, the membrane was incubated with the aforementioned primary antibody, and then the corresponding specific horseradish peroxidase-conjugated secondary antibody was added. The blot was exposed to Hypofilm ECL (GE healthcare, Buckinghamshire, UK), developed, and signal intensity values of the bands were obtained by photodenstometric analysis (Bio-Rad, Laboratories Ltd., Hemel Hempstead, UK).

### Gelatin zymography

Cells were incubated at 37°C in a 5% CO_2_ atmosphere and cultured in 1640 medium without fetal calf serum. The supernatant was collected the next day and centrifuged and stored at −80°C for later use. Then, 10% running gels were prepared with gelatin stock solution (10 mg/ml in H_2_O) added to obtain a concentration of 0.1% (1 mg/ml). The samples (typically 10–25 *μ*l) were applied and the gel was run with 1X Tris-glycine SDS running buffer under the standard running conditions (∼125 V constant voltage). The running was completed when the bromophenol blue tracking dye reached the bottom of the gel. After running, the gel was incubated in the zymogram renaturing buffer and then in zymogram developing buffer. The gel was equilibrated for 30 min at room temperature then put in fresh developing buffer and incubated at 37°C for at least 4 h. The optimal result could be determined empirically by varying the sample load or incubation time. Gels were stained with Coomassie Blue R-250 for 30 min and destained with washing solution (50% methanol, 10% acetic acid). Areas with protease activity appeared as clear bands against a dark blue background where the protease had digested the substrate.

### Statistical analysis

Data are presented as the means ± SD. Parametric testing between two groups was performed by Student’s t-test, and comparison among three or more groups was carried out using one-way ANOVA. For all analyses, P-values <0.05 were regarded as indicative of statistical significance. The statistical analysis was performed using SPSS13.0 for Microsoft Windows (SPSS Inc, Chicago, IL, USA).

## Results

### Establishment and characterization of the VEGF-transfected cells

**Figure 1:**
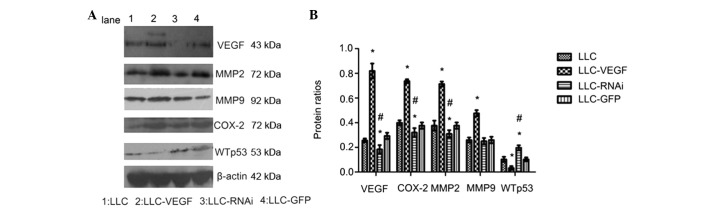
VEGF concentration and its effect on the expression of MMP2, MMP9, COX-2 and WTp53. (A) Equal amount of protein extracts from whole-cell lysates were subjected to western blotting with β-acting serving as an internal control. The results are representative of at least three independent experiments. (B) Bar graphs of VEGF, MMP2, MMP9, COX-2 and WTp53 protein ratios of the four groups. ^*^p<0.05, compared with the LLC group; ^#^p<0.05, compared with the LLC-VEGF group.

To evaluate the biological effect of VEGF on LLC cells, we generated the VEGF gene expression vector. After G418 stable selection, the fluorescence was observed under fluorescence microscopy. Green fluorescence in the LLC-VEGF and LLC-RNAi groups was mainly observed in the cytoplasm, whereas the green fluorescence was scattered in cells in the LLC-GFP group (data not shown). Subsequently, we confirmed the VEGF production by western blotting (Fig. 1A). The expression level of VEGF in the LLC-VEGF group was significant higher (2.19-fold) than that in the control; the VEGF expression was decreased in the LLC-RNAi group, indicating that the VEGF expression was successfully inhibited. This indicated that the cells were transfected successfully.

### Effects of VEGF expression on the protein levels of COX-2, MMPs and WTp53

To further investigate the role of VEGF in the production of MMP2, MMP9 and COX-2, Western blot analysis was used to assess the protein levels. The expression of MMP2, MMP9 and COX-2 was increased 1.89-, 1.83- and 1.84-fold, respectively, in the LLC-VEGF group compared to levels in the control (Fig. 1B), and the expression of MMP2, MMP9 and COX-2 was downregulated 0.82-, 0.96- and 0.8-fold, respectively, in the LLC-RNAi group. Conversely, GFP alone did not influence the protein expression compared with the control. These results demonstrated that the VEGF expression correlated with MMP and COX-2 expression. We also found that VEGF overexpression decreased the expression of wild-type P53, which indicates that VEGF may promote tumor progression via decrease in WTp53 expression.

### VEGF expression correlates with COX-2, MMP2, MMP9 and WTp53 expression

To estimate the correlation between VEGF expression and COX-2, MMP2, MMP9 and WTp53 expression, the correlation coefficient was used to confirm the relationship. There was a significant correlation of VEGF and COX-2, MMP2, MMP9 and WTp53 expression. COX-2, MMP2 and MMP9 were positively correlated with VEGF expression (r=0.984, 0.978 and 0.969, respectively, p<0.01), and WTp53 was negatively correlated with VEGF expression. (r=−0.809, p<0.01)

### Effects of overexpression or underexpression on the growth of Lewis lung cancer cells

**Figure 2:**
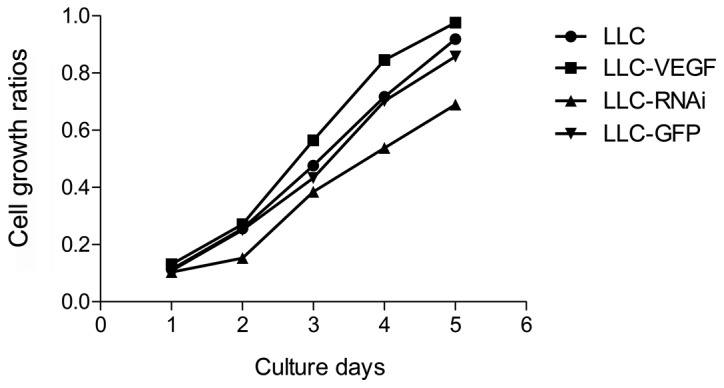
VEGF has no direct effect on the growth of Lewis lung cancer cells *in vitro*. Cells (1×10^4^ cells/ml) were seeded into a 96-well plate, and the number of cells was counted directly every day. The results showed that there was no significant difference in cell growth between the LLC-VEGF or LLC-GFP and LLC groups, but the growth rate was significantly repressed on the 4th and 5th day in the LLC-RNAi group compared with the control (P<0.05).

In order to estimate the effect of VEGF on LLC cell growth, the cell numbers were counted directly each day. The results revealed that there were no changes in the VEGF overexpression group when compared with the control or GFP transfection group, but the growth rate was significantly suppressed on the 4th and 5th day in the LLC-RNAi group as compared with the control (P<0.05) (Fig. 2).

### VEGF promotes LLC cell entry into S phase

**Figure 3:**
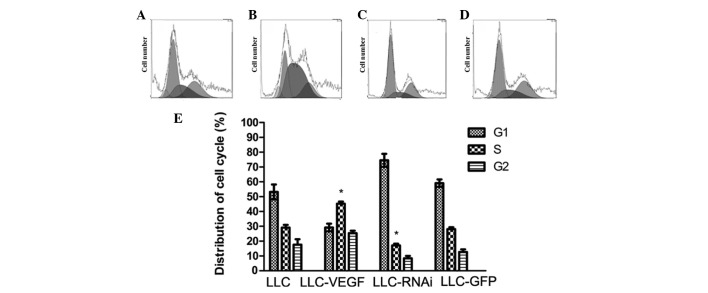
The effect of VEGF on the cell cycle distribution. DNA status was determined by flow cytometry in the cells of the (A) LLC, (B) LLC-VEGF, (C) LLC-RNAi and (D) LLC-GFP groups. (E) Cell cycle distributions of the four groups. ^*^P<0.05; LLC-VEGF or LLC-RNAi group vs. LLC group.

To determine the effect of VEGF on the cell cycle of LLC cells, flow cytometry was performed to determine the cell cycle status. The results showed that there were more cells in the S stage in the LLC-VEGF group (45.3%) compared to the number in the control (29.1%). By contrast, the cell cycle in the LLC-RNAi group was blocked and approximately 74.5% of the cells remained in the G0/G1 stage, and DNA content under the G0/G1 peak was higher, indicating that more cells were undergoing apoptosis. Transfection of GFP alone did not affect cell cycle status (Fig. 3).

### Zymographic detection of MMP activity

**Figure 4:**
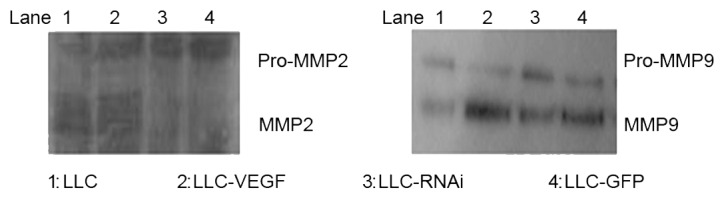
VEGF promotes MMP2 and MMP9 activities in Lewis lung cancer cells. Gelatin zymography was used to detected the MMP activities. The activities of MMP2 and MMP9 were significantly higher in the LLC-VEGF group than in the other groups, but decreased in the LLC-RNAi group.

Zymography was performed to determine the effects of VEGF on MMP2, MMP9 activities. The activities of MMP2 and MMP9 were significantly higher in the LLC-VEGF group than the activities in the other groups, while decreased activities were noted in the LLC-RNAi group (Fig. 4).

## Discussion

This study demonstrated that a certain level of VEGF expression is essential for the growth of LLC cells, and that downregulation of VEGF expression slows the growth of the cells and causes more cells to undergo apoptosis instead of entering the normal cell cycle. Moreover, COX-2, MMP2 and MMP9 was positively correlated to VEGF expression, and WTp53 was negatively correlated to VEGF expression. These findings support our hypothesis.

VEGF, a potent mitogenic and angiogenetic factor, is capable of inducing endothelial cell proliferation, increasing vascular permeability and modifying the status of the extra-cellular matrix or altering gene expression (23–26). Unlike the previous study on endothelial cells, we found that overexpression of VEGF did not exert a marked growth-promoting effect on the LLC cells, whereas downregulation of VEGF slowed the cell growth. Tumor tissue is composed of cancer cells, vascular endothelial cells and infiltrating inflammatory cells (mainly lymphocytes). The discrepancies between our findings and their results may be explained by the fact that they used tumor tissue, whereas we employed cancer cells.

COX-2 is implicated in tumor cell proliferation, resistance to apoptosis, angiogenesis and tumor invasiveness (27). The COX-2-VEGF correlation has been investigated by several groups. Several studies found that VEGF and COX-2 are co-expressed in tumor tissues, and that COX-2 modulates VEGF expression (12–17). An additional study demonstrated that VEGF stimulated the release of COX-2 in endothelial cells by increasing COX-2 transcription and prolonging the COX-2 mRNA half-time (28). However, few data are available on the potential association between VEGF and COX-2 in Lewis lung carcinoma. In the present study, we revealed that elevated VEGF expression induces higher expression of COX-2, whereas downregulation of VEGF leads to lower COX-2 expression. Although the mechanism by which VEGF promotes COX-2 in NSCLC cells is unknown, VEGF increases COX-2 expression in endothelial cells through the P38 MAPK and JNK signal pathways or involves the PKC and NOS pathways (28,29).

Gelatinases (MMP2 and MMP9), two important isoforms in the MMP family, are considered to be closely correlated with tumor invasion and metastasis. Some studies have indicated that there is a close relationship between VEGF and MMP expression. It was demonstrated that VEGF increased the release of MMP2 in brain and ovarian tumor cells (19,30). Moreover, Lee *et al*, using a murine model of asthma, found that inhibition of VEGF receptor downregulated the expression of MMP9 (20). In smooth muscle cells, VEGF promotes MMP9 mRNA transcription and protein activities (31). However, there is a small amount of evidence demonstrating the association among VEGF and MMP2 and MMP9 in Lewis lung carcinoma cells. Our results revealed that VEGF overexpression may increase the production and activity of MMP2 and MMP9. VEGF overexpression-elevated MMP2 and MMP9 expression may explain, in part, the mechanism by which VEGF promotes the invasion of Lewis lung cancer cells.

WTp53 is one of the most intensively studied anti-oncogenes. It acts as a nuclear transcription factor that transactivates genes involved in apoptosis, cell cycle regulation and other important processes (21). It is clear that the wild-type p53 can inhibit angiogenesis, whereas the mutant p53 promotes neovascularization. An *in vitro* study demonstrated that WTp53 downregulated endogenous VEGF mRNA levels and VEGF promoter activity in a dose-dependent manner, whereas mutant forms of p53 did not (22). To date, the association between WTp53 and VEGF remains to be elucidated. The majority of previous studies found that WTp53 expression was negatively associated with VEGF (32), although some studies have yielded discrepant findings (33,34). By examining the tumor samples surgically obtained from 116 esophageal adenocarcinoma patients, Cavazzola *et al* (34) found no correlation between WTp53 protein and VEGF expression. However, it is unclear as to whether VEGF exerts any effect on WTp53 expression. To confirm the definite relation between WTP53 and VEGF, we examined WTp53 protein expression in LLC-VEGF cells. Our results revealed that WTp53 protein levels were negatively correlated with VEGF expression.

In our study, we hypothesized that VEGF did not only promote angiogenesis and metastasis and suppress cell apoptosis independently, but also that it was involved in the production of COX-2 and MMPs and inhibition of WTP53 expression. COX-2, MMP2 and MMP9 were positively correlated with VEGF expression, and WTp53 was negatively correlated with VEGF expression.

In conclusion, our results indicate that overexpression of VEGF enhances the growth of Lewis lung cancer cells, stimulates the production of COX-2, MMP2, MMP9 and enhances the functional activities of MMP2 and MMP9 *in vitro*. VEGF expression, however, suppresses the WTp53. These findings may, at least in part, elucidate the manner by which VEGF is implicated in angiogenesis, invasion and metastasis in lung cancer, and provides experimental basis for anti-angiogenic therapy for cancer. Nevertheless, the exact mechanism of the effect of VEGF on COX-2, MMP2, MMP 9 and WTp53 requires further investigation.
